# Effects of Oxygen Partial Pressure and Substrate Temperature on the Structure and Morphology of Sc and Y Co-Doped ZrO_2_ Solid Electrolyte Thin Films Prepared via Pulsed Laser Deposition

**DOI:** 10.3390/ma15020410

**Published:** 2022-01-06

**Authors:** Jennet R. Rabo, Makoto Takayanagi, Takashi Tsuchiya, Hideki Nakajima, Kazuya Terabe, Rinlee Butch M. Cervera

**Affiliations:** 1 Energy Storage and Conversion Materials Laboratory, Department of Mining, Metallurgical and Materials Engineering, University of the Philippines Diliman, Quezon City 1101, Philippines; jrrabo@up.edu.ph; 2 International Center for Materials Nanoarchitectonics (WIPI-MANA), National Institute for Materials Science, Tsukuba 305-0044, Ibaraki Prefecture, Japan; TAKAYANAGI.Makoto@nims.go.jp (M.T.); TSUCHIYA.Takashi@nims.go.jp (T.T.); TERABE.Kazuya@nims.go.jp (K.T.); 3 Synchrotron Light Research Institute, Nakhon Ratchasima 30000, Thailand; hideki@slri.or.th

**Keywords:** co-doped zirconia, zirconia-doped thin films, pulsed laser deposition, solid electrolyte, XPS

## Abstract

Scandium (Sc) and yttrium (Y) co-doped ZrO_2_ (ScYSZ) thin films were prepared on a SiO_2_-Si substrate via pulsed laser deposition (PLD) method. In order to obtain good quality thin films with the desired microstructure, various oxygen partial pressures ($P_{O_{2}})$ from 0.01 Pa to 10 Pa and substrate temperatures (*T_s_*) from 25 °C to 800 °C were investigated. X-ray diffraction (XRD) patterns results showed that amorphous ScYSZ thin films were formed at room substrate temperature while cubic polycrystalline thin films were obtained at higher substrate temperatures (*T_s_* = 200 °C, 400 °C, 600 °C, 800 °C). Raman spectra revealed a distinct Raman shift at around 600 cm^−1^ supporting a cubic phase. However, a transition from cubic to tetragonal phase can be observed with increasing oxygen partial pressure. Photoemission spectroscopy (PES) spectra suggested supporting analysis that more oxygen vacancies in the lattice can be observed for samples deposited at lower oxygen partial pressures resulting in a cubic structure with higher dopant cation binding energies as compared to the tetragonal structure observed at higher oxygen partial pressure. On the other hand, dense morphologies can be obtained at lower $\;P_{O_{2}}$ (0.01 Pa and 0.1 Pa) while more porous morphologies can be obtained at higher $P_{O_{2}}$ (1.0 Pa and 10 Pa).

## 1. Introduction

Zirconia-based solid oxide electrolyte is one of the most widely used solid electrolytes for solid oxide electrochemical cells (SOEC). It is typically doped with a trivalent cation such as yttrium to create oxygen vacancies and to stabilize the cubic structure. Yttria-stabilized zirconia (YSZ) is the typical zirconia-based solid electrolyte used for SOECs due to its high oxide ion conductivity at elevated temperatures [1,2,3,4]. Scandia-stabilized zirconia (ScSZ), on the other hand, is also used as a solid electrolyte due to its remarkable higher ionic conductivity, almost 3x, as compared to YSZ; however, the high conductivity of the cubic phase ScSZ is limited to a narrow temperature range and with the cubic phase structural stability dependent on doping concentration and temperature [5,6,7,8]. In order to circumvent these concerns and to improve the solid electrolyte properties and performance, fabrication into thin films and co-doping are some of the promising solutions.

Co-doping of scandium and yttrium into zirconia (ScYSZ) has been reported in the literature [8,9,10,11,12,13]. The incorporation of Y^3+^ and Sc^3+^ as co-dopants into ZrO_2_ structure can either stabilize the cubic/tetragonal phase to achieve a better or desired property such as higher conductivity as compared to single doping [8,9,10,11] and can provide good mechanical and thermal stability desirable for certain applications [14]. On the other hand, the fabrication of solid electrolytes into thin films may further increase the conductivity and is crucial for the development of a micron-sized SOEC system (μ-SOEC). ScYSZ thin film showed higher ionic conductivity (1.2 × 10^−1^) at 700 °C (0.7 eV activation energy) as compared to bulk solid electrolytes [8,13,15].

Vapor phase deposition methods are very useful in developing thin films such as nanometer-scale electrode-electrolyte systems. Among these methods, pulsed laser deposition (PLD) has been used for the fabrication of electrolyte thin films with lower ohmic resistance and high-quality films with good morphological features. Such thin films can be attained by simply tuning the stoichiometry of the PLD target material and different deposition parameters [16,17]. Most papers have focused on the fabrication of YSZ [18,19,20,21,22,23,24,25] and ScSZ [6] via PLD and very limited reports on PLD deposited co-doped ScYSZ thin films.

The crystal structure, phase stability, and morphology are important factors that affect the properties such as the conductivity of solid electrolytes and other properties desirable for specific applications. Cubic YSZ has high ionic conductivity; however, its polymorph, tetragonal phase, although has lower conductivity, shows interesting high mechanical strength and hardness particularly also useful for solid oxide electrochemical cells applications [14,26,27,28]. For ScSZ, the reported ionic conductivity trend, high to low, is cubic > tetragonal > rhombohedral > monoclinic (with tetragonal phase), respectively [29]. In addition, ScSZ denser films were prepared also at low oxygen partial pressure using low-pressure plasma spray to achieve higher oxygen ion transport performance [30]. In the fabrication of thin films using PLD, the resulting crystal structure and morphology of the deposited thin films, which directly affect the electrical properties of the thin films, are dependent on the parameters employed during deposition [24,25]. For YSZ, deposited thin films show the evolution of microstructure from porous to dense thin films. High deposition temperature and lower oxygen partial pressure resulted in dense microstructure and less dense at increasing oxygen partial pressure. Dense microstructure thin film has high oxygen ion conductivity while porous thin films have low oxygen ion conductivity but have high protonic conductivity [25,31,32]. For scandium and yttrium co-doped zirconia or ScYSZ thin films, very limited information or none so far has been reported on the effects of PLD deposition parameters on the properties of ScYSZ solid electrolyte thin films. Hence, it is important to explore and investigate ScYSZ thin films fabricated via PLD.

In this study, in order to tailor and to provide an understanding of the quality of Sc and Y co-doped ZrO_2_ thin films produced via PLD, the effects of different PLD deposition parameters such as oxygen partial pressure and substrate temperature on the crystal structure and morphology of Sc-Y co-doped ZrO_2_ deposited thin films were investigated. The properties of prepared thin films were analyzed using X-ray diffraction (XRD), Raman spectroscopy, scanning electron microscopy (SEM), and Photoemission spectroscopy (PES).

## 2. Materials and Methods

The PLD ScYSZ target material, scandium and yttrium co-doped zirconia having a chemical composition of Zr_0.4_Y_0.8_Sc_0.8_O_1.92_, was prepared via solid state reaction method using ZrO_2_ (5–25 nm, 97.2%, EM Futur, Castellon, Spain), Sc_2_O_3_ (99.9% Sigma-Aldrich, Inc., St. Louis, MO, USA), and Y_2_O_3_ (<50 nm, Sigma-Aldrich, Inc., St. Louis, MO, USA) powders. The as-calcined powder was pelletized using uniaxial pressing and finally sintered at 1400 °C in ambient condition and was used as target material for PLD. ScYSZ thin films were ablated, with a 30 mm distance, on Si with native SiO_2_ oxide substrate using Nd^3+^: YAG laser (LOTIS TII (LS-2137U), *λ* = 266 nm), Tokyo Instruments, Inc., Tokyo, Japan using laser energy of 38 J and a repetition rate of 10 Hz. The pulse duration was 10 ns, the area of the beam was 9.8 × 10^−^³ cm^2^ and a plano-convex lens was used with a focal length of 400 mm. The as-deposited ScYSZ thin films were prepared and investigated using various PLD substrate temperatures (*T_s_* = 25 °C, 200 °C, 400 °C, 600 °C, 800 °C) and oxygen partial pressures ($P_{O_{2}}$ = 0.01 Pa, 0.1 Pa, 1.0 Pa, and 10 Pa).

The crystal structure of the as-deposited ScYSZ thin films was determined using Rigaku SmartLab X-ray Diffractometer (Rigaku Corporation, Tokyo, Japan). XRD analysis was performed using a 2-theta measurement (through NIMS-Namiki Foundry Facility, Tsukuba, Japan), equivalent to the grazing technique used for thin-film characterization. Scanning was obtained with CuKα (*λ* = 1.54 nm), resolution of 0.01, and 2θ scan range from 20° to 80° with 5°/min scan rate. To further investigate the crystal structure and polymorphs of ScYSZ thin films, JASCO NRS-5100 Laser Raman spectrometer (Jasco International Co., Ltd., Tokyo, Japan) was used. Raman shift of the as-deposited thin films was obtained with the wavenumber ranges from 100 cm^−1^ to 1200 cm^−1^ at room temperature. Field Emission-Scanning Electron Microscopy (FE-SEM) S-4800 (Hitachi High-Tech., Corp., Tokyo, Japan) was used to observe the morphological features of as-deposited ScYSZ thin films. Before the sample measurements, Pt films were sputtered on the surface of ScYSZ film for 20 s. Both the surface structure and the cross-sectional images were captured. Photoemission Spectroscopy (PES) was performed with Beamline 3.2Ua Photoelectron spectroscopy at the Synchrotron Light Research Institute, Nakhon Ratchasima, Thailand. The electron energy was analyzed by CLAM2 (Thermo VG Scientific, England, UK). The photon energy was 600 eV and the total energy resolution was about 2 eV. The base pressure was 2 × 10^−8^ Pa.

## 3. Results and Discussions

### 3.1. X-ray Diffraction Analysis

**Figure 1:**
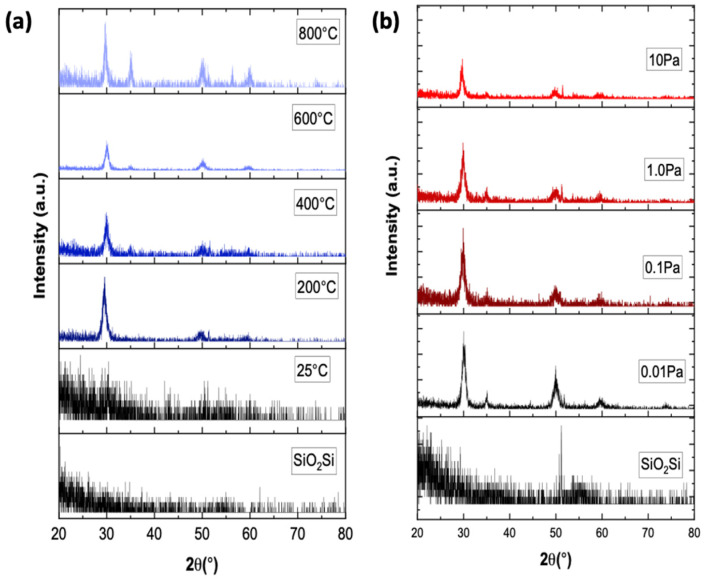
XRD stacked pattern. (**a**) Diffraction peaks of ScYSZ thin film on SiO_2_-Si prepared with various substrate temperatures (*T_s_* = 25 °C, 200 °C, 400 °C, 600 °C, and 800 °C) at $P_{O_{2}}$ = 0.1 Pa and (**b**) XRD of ScYSZ thin film on SiO_2_-Si prepared at *T_s_* = 600 °C, with various oxygen partial pressures, $P_{O_{2}}$ = 0.01 Pa, 0.1 Pa, 1.0 Pa and 10 Pa.

Figure 1 shows the diffraction peaks of polycrystalline as-deposited ScYSZ thin films using different deposition parameters: *T_s_* and $P_{O_{2}}$. Figure 1a shows that at increasing deposition temperature, crystallized ScYSZ thin films were obtained. The as-deposited ScYSZ thin films at about 25 °C or room temperature (RT) is amorphous and crystallization started at 200 °C, then well-crystallized peaks can be observed from 600 °C to 800 °C. The diffraction peaks at *2θ* ≈ 30° (111), 35° (200), 50° (220), 60° (311), 63° (222), and 75° (400) can be indexed and attributed to cubic ZrO_2_-phase (ICSD No. 00-078-1808). This result is similar to the diffraction peaks measured for the target pellet and is observed to agree with the previous studies in [17,24,25] on YSZ thin films on SiO_2_-Si substrate. On the other hand, the effect of oxygen partial pressure on the crystal structure of ScYSZ thin films is shown in Figure 1b. As shown in the XRD stacked patterns, similar peaks can be observed for the different oxygen partial pressures; however, more crystalline or high-intensity peaks are observable for the oxygen-deficient environment or lower oxygen partial pressure, $P_{O_{2}}$ = 0.01 Pa and 0.1 Pa, as compared to higher oxygen partial pressures peak intensities.

### 3.2. Raman Spectroscopy

**Figure 2:**
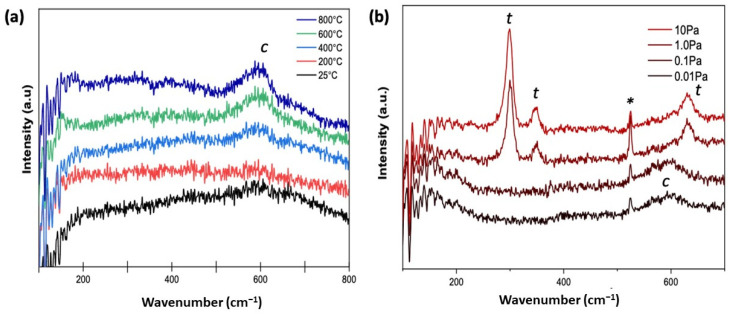
Raman spectra of (**a**) as-deposited ScYSZ thin films on PtTi-SiO_2_-Si substrate with varying substrate deposition temperatures, *T_s_* = 25 °C, 200 °C, 400 °C, 600 °C, and 800 °C, with constant $P_{O_{2}}$ = 0.1 Pa; and (**b**) with various oxygen partial pressures, $P_{O_{2}}$ = 0.01 Pa, 0.1 Pa, 1.0 Pa and 10 Pa, at *T_s_* = 600 °C. (* SiO_2_ substrate).

Figure 2a depicts the Raman spectra of the as-deposited ScYSZ thin films. When deposited at increasing deposition temperature, ScYSZ thin films show mostly cubic (**c**) phase with distinct Raman shift at approx. 600 cm^−1^. On the other hand, while the XRD results revealed only cubic phases for all deposited samples, due to sensitivity of Raman spectroscopy to local structure and disordering as compared to XRD, there is an observed transition from cubic phase to tetragonal (**t**) phase for ScYSZ thin films deposited with increasing oxygen partial pressure as shown in Figure 2b.

As revealed in Figure 2b, in an oxygen-deficient environment or low oxygen partial pressures, $P_{O_{2}}$ = 0.01 Pa and $P_{O_{2}}$ = 0.1 Pa, only a single Raman band at around 600 cm^−1^ was observed suggesting a cubic ZrO_2_ fluorite structure [28,33]. However, at increasing oxygen partial pressure, from $P_{O_{2}}$ = 1.0 Pa to $P_{O_{2}}$ = 10 Pa, Raman bands at approx. 298 cm^−1^, 350 cm^−1^, 630 cm^−1^ are observable which can be attributed to coupling of Zr-O′ stretching, O(O′)-Zr-O(O′) bending, and Zr-O stretching vibrational modes, respectively [10,28,34]. The observed Raman band at around 524 cm^−1^ is attributed to vibrational mode coming from the SiO_2_ substrate which is more distinct at high oxygen partial pressures.

### 3.3. Microstructural Properties

**Figure 3:**
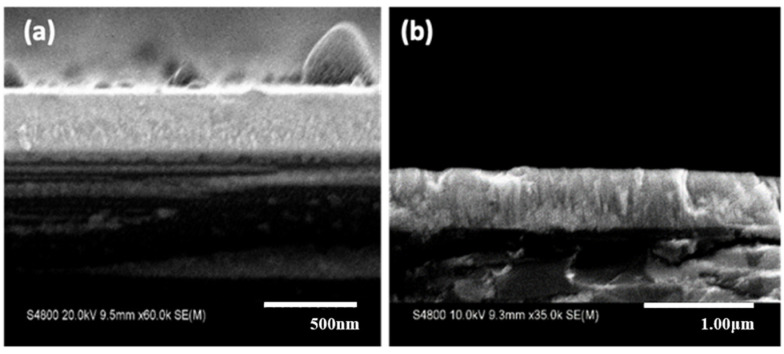
Cross-sectional SEM images of ScYSZ prepared at (**a**) *T_s_* = 25 °C (**a**,**b**) *T_s_* = 800 °C, with $P_{O_{2}}$ = 0.1 Pa, 3 h deposition time on SiO_2_-Si substrate.

Figure 3 shows the cross-sectional SEM images of the deposited thin films for *T_s_* = 25 °C (Figure 3a) and *T_s_* = 800 °C (Figure 3b) with oxygen partial pressure of $P_{O_{2}}$ = 0.1 Pa and deposition rate of about 70 nm/h. Although both substrate temperatures showed a dense morphology, a more crystalized columnar structure can be observed at high substrate temperature.

**Figure 4:**
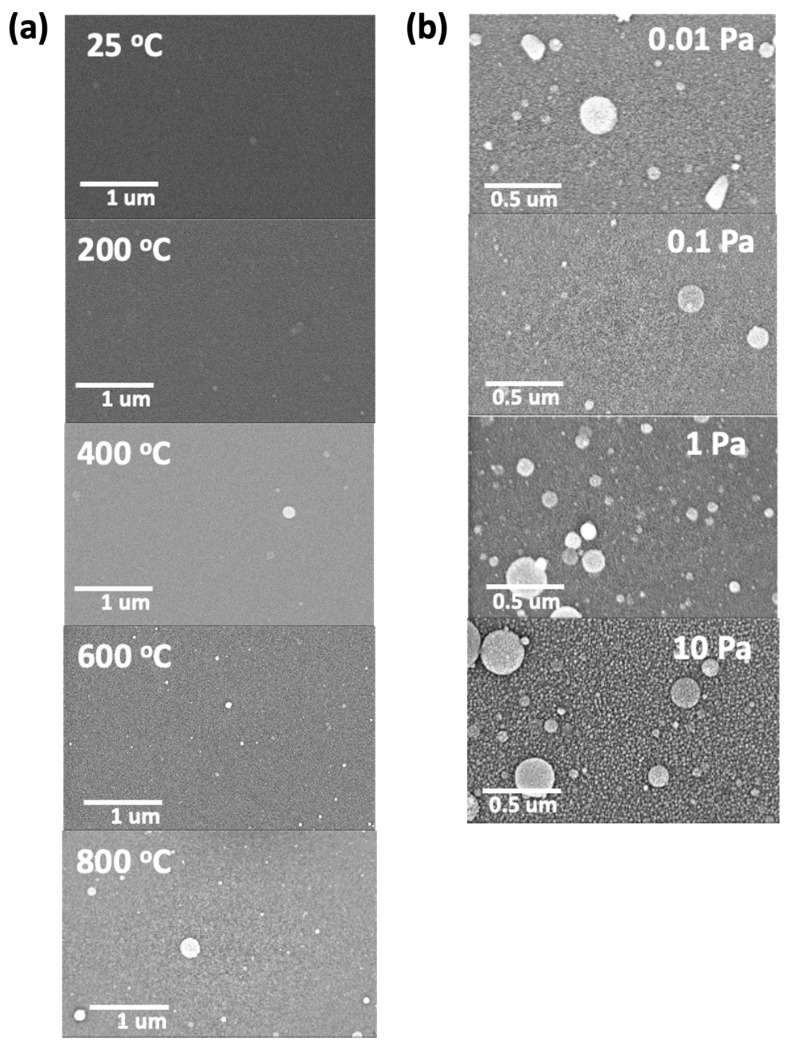
SEM surface morphology images of ScYSZ thin film on SiO_2_-Si substrate prepared (**a**) at various substrate temperatures with constant $P_{O_{2}}$ = 0.1 Pa; and (**b**) at various oxygen partial pressures ($P_{O_{2}}$) at 600 °C.

The surface morphologies of the deposited thin films at different substrate temperatures and oxygen partial pressures are shown in Figure 4a,b, respectively. As can be observed in Figure 4a, the images revealed dense and crack-free surfaces, with the presence of droplets or circular agglomerates that increases at increasing deposition temperature. These agglomerated droplets are ScYSZ particles which are characteristic deposits for oxide films prepared using Nd:YAG laser system in PLD [20,33,35]. On the other hand, Figure 4b showed the SEM surface images of the as-deposited thin film on SiO_2_-Si substrate at various oxygen partial pressure. At increasing oxygen partial pressure, more porous and rough surface microstructures are observable. This effect on the surface morphology using higher oxygen partial pressure during deposition is consistent with reported works on the microstructure of ZrO_2_ thin films [19,24,25,36].

### 3.4. Photoemission Spectroscopy (PES)

**Figure 5:**
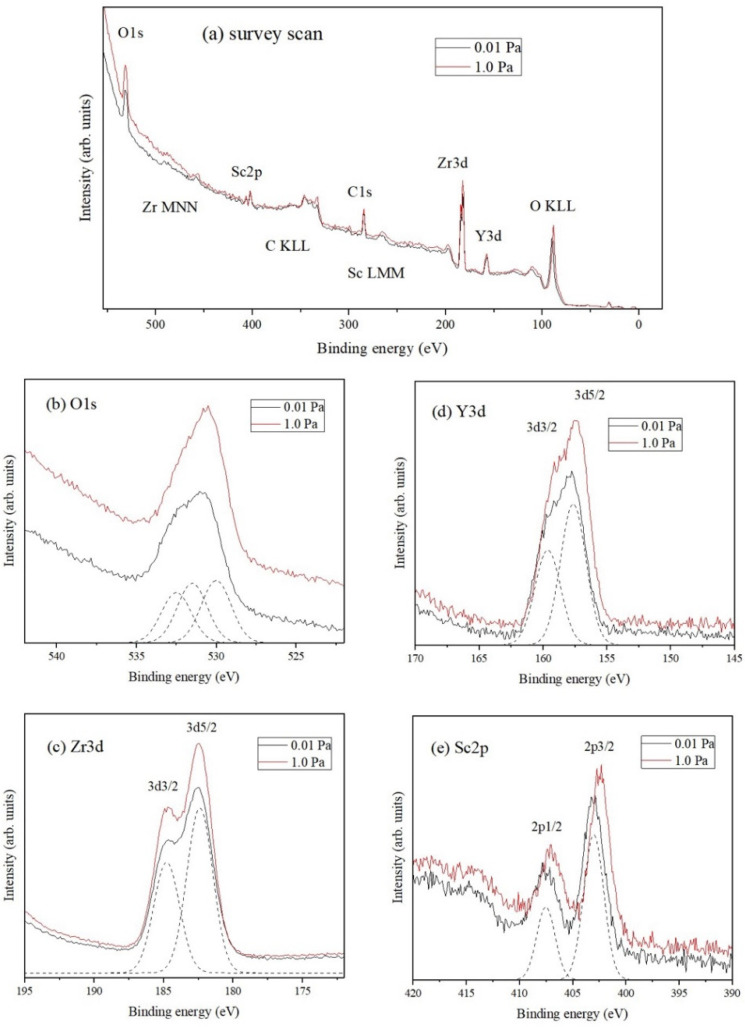
(**a**) PES wide scan spectra of ScYSZ thin film on SiO_2_-Si substrate prepared at *T_s_* = 600 °C with $P_{O_{2}}$ = 0.01 Pa and $P_{O_{2}}$ = 1.0 Pa, and (**b**–**e**) comparative stack spectra with $P_{O_{2}}$ = 0.01 Pa and 1.0 Pa showing the (**b**) O 1s, (**c**) Zr 3d, (**d**) Y 3d, and (**e**) Sc 2p core levels.

The co-doping of Sc and Y in ZrO_2_ and its effect on the resulting thin film structure was verified by the presence of the element’s core levels and with binding energies using PES analysis [37,38,39] and the results are shown in Figure 5. Figure 5a shows the wide scan spectra for ScYSZ thin films deposited with $P_{O_{2}}$ = 0.01 Pa and $P_{O_{2}}=$ 1.0 Pa with binding energies and peaks identified for O 1s, Sc 2p, Zr 3p and Zr 3d, Y 3p and Y 3d, and O_KLL_ core levels. The binding energy of the spectra was referenced from C 1s at 284.6 eV. PES peaks position for Zr 3d is at 181 eV to 183 eV which corresponds to the Zr^4+^ valence state. On the other hand, for Y 3d, the peak position is at around 158 eV for Y^3+^ valence state [40,41] and the peaks for Sc 2p are at around 402 eV to 403 eV for the Sc^3+^ valence states [38,42]. For the O 1s core levels, the peaks are expected at binding energies around 530 eV to 532 eV for lattice oxygen and hydroxyl [39], respectively, and O KLL is expected at 92 eV. The narrow scans for different core levels are shown in Figure 5b–e.

Figure 5b shows the PES stacked spectra of the O 1s core level for ScYSZ thin films deposited at $P_{O_{2}}$ = 0.01 Pa and $P_{O_{2}}=$ 1.0 Pa. The PES spectrum was observed to be slightly asymmetric for both $P_{O_{2}}$, 0.01 Pa and 1.0 Pa. A sample deconvolution of the peaks, for the 0.01 Pa spectrum, is also shown. As revealed, O 1s can be deconvoluted or fitted into three peaks centered at 530.0, 531.5, and 532.5 eV, for both $P_{O_{2}}$. These binding energies are typical values for O1s lattice oxygen (oxygen bonded with Zr and/or Sc/Y), hydroxyl, and weakly adsorbed oxygen/H_2_O [42,43,44,45]. The PES stacked spectra of Zr 3d, Y 3d, and Sc 2p for the two oxygen partial pressures are shown in Figure 5c–e, respectively. Spin-orbit splitting can be observed from these spectra. Figure 5c shows the PES spectra of Zr 3d consisting of Zr 3d_5/2_ and Zr 3d_3/2_, corresponding to the Zr^4+^ valence state, with binding energies located at 182.4 eV and 184.8 eV for $P_{O_{2}}=$ 1.0 Pa, respectively. On the other hand, Figure 5d shows the PES spectrum of Y 3d that is slightly asymmetric which consists of Y 3d_5/2_ and Y 3d_3/2_ with binding energies located 157.2 eV and 159.2 eV for $P_{O_{2}}=$ 1.0 Pa corresponding to the Y^3+^ valence state. The PES spectrum of Sc 2p consisting of Sc 2p_3/2_ and Sc 2p_1/2_ is shown in Figure 5e, with binding energies located at 402.4 eV and 406.9 eV ($P_{O_{2}}=$ 1.0 Pa), corresponding to Sc^3+^ valence state.

The effects of increasing $P_{O_{2}}$ in the ScYSZ thin film deposition are observable by the slight shifting in the binding energies of the dopants (Y 3d/Sc 2p) core levels. The Y 3d peak shifts from 157.6 eV to 157.2 eV while Sc 2p3/2 peak shifts from 403.0 eV to 402.4 eV as oxygen pressure increases from $P_{O_{2}}$ = 0.01 Pa to $P_{O_{2}}$ = 1.0 Pa. This means that ScYSZ thin films deposited at $P_{O_{2}}\;$ = 0.01 Pa suggested a higher concentration of oxygen vacancy as compared with ScYSZ thin films deposited at $P_{O_{2}}$ = 1.0 Pa [39]. It can be said that the lower oxygen partial pressure induces oxygen defects or vacancies; however, as the oxygen partial pressure increases from 0.1 Pa to 1.0 Pa, the lattice oxygen increases. These suggest that the dopants were reduced when oxygen pressure was increased. On the other hand, from the results in this study, the oxygen partial pressure does not crucially affect the Zr 3d peaks. However, for the dopant cations, Y 3d and Sc 2p, the binding energy decreases as the oxygen partial pressure increases. It can be said that at higher oxygen partial pressures, the oxygen vacancy may be filled preferentially in the vicinity of the dopant thereby significantly affecting the binding energies of Sc/Y while Zr, in general, may have no drastic changes in its environment. From the fitting data, the Zr is only slightly affected with a very small binding energy changed, about 0.02 eV difference due to the local structural change effect also with the nearest dopant. Further study is needed to support the coordination numbers of these cations and the theoretically expected formation of non-lattice oxygen with the doping that affects the binding energy. It can be said though, in this study, that the suggested presence of oxygen vacancies may be one of the reasons for the structural transition from cubic to tetragonal phase at lower to higher oxygen partial pressures, as also supported in the local structural phase transition observed from the Raman analyses.

## 4. Conclusions

Thin films of scandium and yttrium co-doped zirconia (ScYSZ) were successfully prepared on SiO_2_-Si substrate using PLD under varying oxygen partial pressures ($P_{O_{2}}$ = 0.01 Pa, 0.1 Pa, 1.0 Pa, and 10 Pa) and substrate temperatures (*T_s_* = 25 °C, 200 °C, 400 °C, 600 °C, 800 °C. From the XRD analysis, an amorphous film was produced at room temperature; however, a polycrystalline cubic-phase ScYSZ can be achieved at lower oxygen partial pressures ($P_{O_{2}}$ = 0.01 Pa and 0.1 Pa) and increasing substrate temperatures (*T_s_* = 200 °C, 400 °C, 600 °C, 800 °C). The cubic structure at low oxygen partial pressure was supported by the observed Raman shifts in the Raman spectra. However, at high oxygen partial pressures ($P_{O_{2}}$ = 1.0 Pa and 10 Pa), a tetragonal phase can be observed from the Raman spectra. On the other hand, SEM images revealed dense and crack-free surface morphologies with particle droplet-like agglomerates achieved at lower oxygen partial pressures ($P_{O_{2}}$ = 0.01 Pa and 0.1 Pa), and rough surface and porous morphology at increasing oxygen partial pressures ($P_{O_{2}}$ = 1.0 Pa and 10 Pa). Furthermore, PES spectra revealed the shifting of the dopant cations core level peaks with the increase in oxygen partial pressure from higher to lower binding energy.

## Data Availability

Not applicable.
